# Maryland and District of Columbia Dental Association

**Published:** 1878-11

**Authors:** 


### EDITORIAL, ETC.

---

*Maryland and District of Columbia Dental Association.*—The above Association held its Fourth Annual Meeting in Washington, last month. The work of the Association has been committed to Sections, each member thereby being able thus to follow the bent of his own mind, year after year, of a single line of investigation. It is thought that good will result from this arrangement.

If it be not out of place, we would suggest to the Sections that original investigation is of all things most important, and is always interesting. A re-statement of the views of others is hardly what is wanted, nor is a resumé of what has been developed in this department or the other so much to be desired as some original examination of a question. It is, as they say in Germany, not so important to know what we have *acquired*, as what we have *done;* and it is only important to know what others have done as a means of guiding our own investigations, and aiding our own search after truth.

The desire which prompts some to speak on all subjects, is, beyond a doubt, in the way of the felicitous progress of the work of an Association. Impromptu remarks are not always pertinent. As well had one write for all the sections, or speak on all papers read from them; and reflections of the paper read might well be omitted.

This is written in no sense of intended harsh criticism of the proceedings of the above Association, which were in the main, harmonious, but in the hope that assistance may be given to deliberations which are more and more important from year to year, as the scientific aspect of dentistry is growing more apparent. Earnest thought and careful investigation, conducted in the light of science, are making some of the members of the dental profession marked as men of whose attainments any profession might well be proud; and it is doubtful if we are in a position or a condition to criticise them until we have pursued somewhat the same line of investigations which brought about these results.

The paper on the Morphology of the Blood, by Dr. E. Cutler, of Boston, was, of all those read, by far the most interesting. It deserves a close reading; and we are glad to read that its repetition in Baltimore was before a large audience. Dr. Cutler has been ably assisted by Dr. G. B. Harriman, a dentist of Boston, in these investigations. Dr. J. J. Caldwell, of Baltimore, also contributed largely to the success of the Association by the able paper on Anæsthesia, and on Alcoholism and Syphilis.

